# Influence of taste stimulation on sucking pressure in newborn infants at term

**DOI:** 10.1590/2317-1782/20212021002

**Published:** 2022-01-12

**Authors:** Fernanda Segala, Geovana de Paula Bolzan, Marlove Duarte Nascimento, Daniela da Silva Gonçalves, Amanda Melchior, Marcus Vinícius Marques de Moraes, Angela Regina Maciel Weinmann

**Affiliations:** 1 Programa de Pós-graduação em Distúrbios da Comunicação, Universidade Federal de Santa Maria – UFSM - Santa Maria (RS), Brasil.; 2 Departamento de Fonoaudiologia, Universidade Federal de Santa Maria – UFSM - Santa Maria (RS), Brasil.; 3 Departamento de Fisioterapia, Universidade Regional de Blumenau – FURB - Blumenau (SC), Brasil.; 4 Departamento de Fisioterapia, Hospital Universitário de Santa Maria, Universidade Federal de Santa Maria – UFSM - Santa Maria (RS), Brasil.; 5 Programa de Residência Multiprofissional, Hospital Universitário de Santa Maria, Universidade Federal de Santa Maria – UFSM - Santa Maria (RS), Brasil.; 6 Departamento de Fisioterapia, Universidade Regional de Blumenau – FURB - Blumenau (SC), Brasil.; 7 Departamento de Pediatria e Puericultura, Universidade Federal de Santa Maria – UFSM - Santa Maria (RS), Brasil.

**Keywords:** Sucking Behavior, Feeding, Taste Perception, Newborn, Human Milk

## Abstract

**Purpose:**

To verify the influence of a taste stimulus on the suction pressure, during the non-nutritive sucking (SNN), in newborns, healthy and with weight appropriate to the gestational age.

**Methods:**

Quasi-experimental study of the non-randomized clinical trial type with a convenience sample of 60 newborns (NB), 30 allocated in the study group (EG) and 30 in the control group (CG). The NB were evaluated for sucking pressure during the SNN in a pacifier. For the EG, a gustatory stimulus was added to the pacifier, moistened with colostrum. The CG did not receive any stimulus, other than the pacifier itself. The average, minimum and maximum pressures were measured with the equipment S-Flex®.

**Results:**

The SG presented mean and maximum sucking pressure significantly higher than the CG. In addition, there was a statistically significant difference between the groups for the second measurement of mean sucking pressure.

**Conclusion:**

The results showed that the NB of the SG presented sucking pressures, average and maximum, significantly higher, when compared to the CG. The use of a taste stimulus associated with SNN modified the sucking pressure and seems to enhance oral skills.

## INTRODUCTION

The sucking reflex is crucial for newborns and emerges in intrauterine life, maturing between the 32nd and 34th week of gestational age^(1)^. At approximately 12 gestational weeks, the fetus displays suction behaviors, and at 20 weeks, can open and close the mouth in cycles of organized bursts and regular pauses^(2)^.

The sucking reflex allows the newborn to ingest milk immediately after birth. Sucking behavior can also have a calming effect during painful procedures, contribute to the development of the stomatognathic system, and stimulate oral perception and environmental exploration^(3,4)^.

In addition to adequate suction and the coordination between sucking, swallowing, and breathing^(5)^, behavioral organization is also a major indicator of successful oral feeding in newborns^(6,7)^. The maintenance of a state of alertness during oral feeding allows the newborn to familiarize themselves with all oral, auditory, vestibular, haptic, and kinesthetic sensations and stimuli associated with this activity, promoting a pleasant, efficient, and safe feeding experience^(8-10)^.

The evaluation of non-nutritive sucking (NNS) behavior is involved in the assessment of oral feeding readiness and the need for oral-motor intervention, both of which are crucial for the maturation of oral feeding skills in infants with difficulties in these domains, especially those born preterm^(1,11-13)^. As a result, researchers have introduced several tools to analyze both behavioral and sensory aspects of NNS^(3,9,14-17)^.

One such tool is the S-FLEX® device, which can quantify the pressure of NNS in newborns^(18)^, increasing the precision of this assessment. The device has all the necessary features for use in routine neonatal examination, including portability, ease of handling, and the ability to be cleaned and sterilized for use in hospital settings. The S-FLEX® also ensures the environmental safety of newborns as it does not expose them to electrical currents or magnetic fields.

The reliability and reproducibility of the S-FLEX® in the assessment of NNS pressure in newborn infants has been determined in previous studies which have demonstrated its use and scientific applicability^(19)^. In the search for increasingly objective and quantitative measures of NNS, some studies have introduced the use of taste stimulation in these assessments^(6,8,20-22)^.

Taste stimulation appears to increase the frequency and regularity of sucking, contributing to the constancy of movement of the orbicular muscles of the mouth and the sensory-motor system^(23)^. The availability of additional stimuli increases the pleasure and satisfaction experienced by the newborn, promoting psychological, affective, and biological processes that make for a more effective and reliable assessment of feeding performance^(14,17,19,24)^.

In the present study, we examined the effect of colostrum as a gustatory stimulus on the assessment of NNS in newborns. Our aim was to contribute to the clinical practice of professionals involved in the assessment of NNS in newborns by analyzing the influence of taste stimulation on sucking pressure during the quantitative assessment of NNS in full-term, healthy newborns before discharge from the maternity ward.

## METHODS

### Study design

Quasi-experimental, non-randomized clinical trial.

### Sample

The sample was recruited by convenience and consisted of 60 newborns admitted to the maternity ward of the University Hospital of Santa Maria (HUSM) from April to November 2019. Participants were divided into two groups through non-random assignment. The 30 infants in the experimental group (EG) were given taste stimulation during the assessment of NNS while the 30 infants in the control group (CG) were not.

### Inclusion criteria

Eligible participants consisted of full-term newborns (with gestational age ≥ 37 weeks), up to the third day of life, who were clinically stable, on exclusive breastfeeding, with adequate weight for gestational age, and whose parents and/or legal representatives provided written informed consent to their participation in the study.

### Exclusion criteria

Newborns with congenital head and neck, neurological, or cardiac malformations were excluded from the study, as were those with genetic syndromes or presenting with respiratory and/or clinical instability at the time of assessment.

### Ethical aspects

The study was approved by the Research Ethics Committee of the Federal University of Santa Maria, under protocol number 11155312.7.00005346. All participants were authorized to enter the study by their legal guardians who signed an informed consent form.

### Procedures

First, the hospital admission forms were reviewed for the identification of eligible participants who met the aforementioned inclusion criteria. The parents and/or legal guardians of eligible participants were then given an informed consent form, and all newborns who received parental consent to participate were included in the study. Subsequently, the following birth-related information was collected: identification, date and time of birth, gestational age and weight at birth, 1- and 5-minute Apgar scores, type of delivery, and adequacy of intrauterine growth.

The assessment of sucking pressure during NNS (main variable of study) was performed by a trained examiner, with the newborn in an alert behavioral state having last been fed at least one hour before the assessment.

In both the EG and CG, sucking pressure was measured using the S-FLEX® (Todmed) device. One full minute of active suction was analyzed for each participant. Sucking pressure was recorded using a pacifier. All mothers were informed that the pacifier would be used as an assessment tool but were also told of the risks associated with pacifier use beyond the moment of assessment, especially concerning early weaning and the development of the stomatognathic system. As such, we do not believe that the use of a pacifier during the assessment increased the likelihood that mothers would subsequently offer pacifiers to their children.

Data collection began with the manual expression of a small amount of colostrum which was stored in a plastic cup. This stage was conducted before the assessment of EG participants only. Prior to the assessment itself, all infants in the EG and CG were also given time to adapt to the pacifier. The only difference between groups was the addition of the taste stimulus to the pacifier in the EG (as previously described).

Subsequently, the newborn was placed in the examiner’s lap, in a horizontal position with the head and trunk elevated. The examiner used the pacifier to gently stimulate the rooting reflex and encourage the newborn to open their mouth so that the pacifier could be inserted into the oral cavity. The examiner supported the pacifier with their thumb and index finger, offering stability but no resistance.

If the newborn pushed out the pacifier in an attempt to spit it out, the examiner released it and reinserted it into the oral cavity to resume the evaluation. Infants were allowed to suck on the pacifier until the end of the assessment. We did not establish a maximum number of times for the pacifier to be reinserted into the infants’ oral cavity, and this variable was not considered during data analysis. However, at the slightest sign of stress from the infant, including crying or demonstrations of fatigue, data collection was interrupted and only resumed when the newborn showed signs of comfort.

The recording of NNS pressure only began after the infant had adapted to the shape and texture of the pacifier. For the addition of the taste stimulus, the examiner dipped a gloved finger in the colostrum and used it to touch the pacifier. The volume of colostrum involved in this procedure was not measured, as the amount of colostrum required was quite small, amounting to approximately one drop. The moistened pacifier was immediately offered to the infant. As soon as the newborn adapted to the pacifier and began to display effective sucking behaviors, the assistant examiner was asked to activate the display of the S-FLEX® to record the sucking pressure. The pacifier was considered to be loosely held in the mouth when the recorded pressure was zero. Since the S-FLEX® has a maximum storage time of 22 seconds, three measurement periods were completed for each infant to obtain a full minute of recordings. The maximum duration of the assessment was ten minutes. Lastly, to register and record the measurements, the assistant examiner analyzed three records and identified those with the clearest outline to be used in subsequent analysis.

At the end of the assessment, all material was thoroughly cleaned and heat-treated per hospital guidelines.

**Figure 1:**
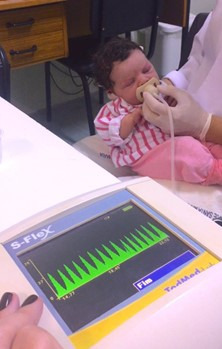
Data collection procedure with an NB, its positioning and support of the pacifier

Figure 1 illustrates the data collection procedure, including the position of the infants in the examiner’s lap and how the pacifier was supported in the oral cavity of the neonate.

NNS pressure was measured using the nationally manufactured S-Flex® device (Todmed). This equipment registers NNS pressure in numerical and graphical formats. The equipment has a touchscreen and can communicate with personal computers through *a* Windows® software interface. It also has a USB port (for *USB* or external hard drives) and a 127 to 240V external power source, which charges a battery that can last up to four hours. Measurements were made in physical units and recorded in mmHg and g/cm^2^.

**Figure 2:**
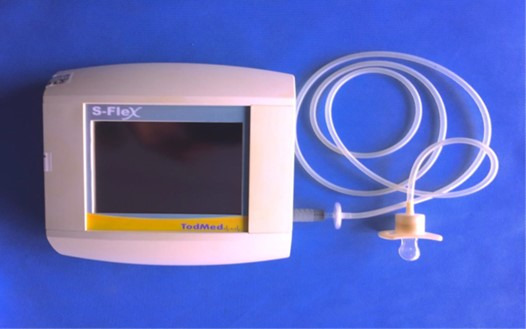
S-FLEX® Equipment

**Figure 3:**
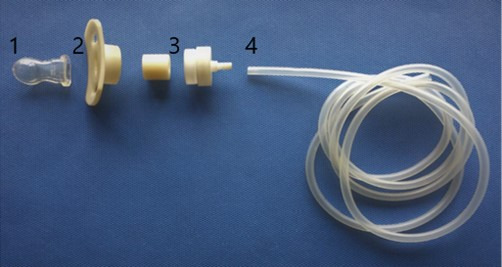
Components of the S-FLEX® equipment

The S-FLEX® contains a pacifier with a small hole attached to a concave, anatomically-shaped ring with a pressure sensor. The pacifier is attached to the device by a 1.5m long tube with a thickness of 1.25mm. The pacifier is made of silicone and orthodontically shaped. It was extracted from a Size 1 Oral Fit pacifier from commercial brand NUK®, recommended for infants aged 0 to 6 months. A small orifice was opened at the end of the pacifier with a 1014 diamond bur (KGS). The materials and components are for individual use and can be cleaned and sterilized through heat treatment with no risk of cross-contamination. The tube that connects the pacifier to the rest of the equipment meets biocompatibility and electrical safety standards, in accordance with hospital biosafety protocols. The S-FLEX® is also light and portable and allows for data to be collected in different places. The S-FLEX® and its components are depicted in Figures 2 and 3, respectively.

Caption: (1) orthodontic tip; (2) concave and anatomical arch; (3) pressure sensor; (4) 1.25 mm × 1.5 m tube

### Data analysis

The data were entered into Excel spreadsheets and analyzed using STATA 10. Continuous variables were expressed as means and standard deviations and categorical variables as percentages. The Shapiro-Wilk test was used to test the normality of distributions. The groups were compared using Student's t-test (continuous variables) and Pearson's Chi-square test (categorical variables). Results were considered significant at p < 0.05.

## RESULTS

The characteristics of the EG and CG are shown in Table 1. In the EG, the mean gestational age and weight at birth were 39 (±1.1) weeks and 3141 (± 262) grams, respectively, while in the CG, the corresponding values were 38.7 (±1.10) weeks and 3132 (±414) grams. All infants in both groups were classified as having adequate weight for gestational age at birth. In the EG, 53.3% of participants were girls and 46.7% were boys. In the CG, most participants were boys (60% boys, 40% girls). In both groups, the mean Apgar scores at 1 and 5 minutes were greater than seven. Despite the lack of random assignment, the characteristics of the two groups did not significantly differ from one another.

**Table 1:** Characteristics of studied full-term newborn infants

	Presence of gustatory stimulus in NNS		
	EG N=30	CG N=30	p
Weight at birth (g)	3141 ± 262	3132 ± 414	0.46^1^
GA at birth (wk)	39 ± 1.1	38.7 ± 1.1	0.17^1^
Gender (% (N))			
Male	46.7 (14)	60 (18)	0.30^2^
Female	53.3 (16)	40 (12)	
Delivery type			
Vaginal birth	53.3 (16)	73.3 (22)	0.10^2^
Cesarean birth	46.7 (14)	26.7 (8)	
1st-minute Apgar	8.6 ± 1.3	8.8 ± 1.3	0.22^1^
5th-minute Apgar	9.7 ± 0.5	9.8 ± 0.4	0.19^1^
Days of life assessment	2 ± 1.26	1.67 ± 0.71	0.261^1^

1 Student's t-test;

2 Pearson's Chi-square

Caption: EG = experimental group; CG = control group; NNS = non-nutritive suction; GA = gestational age; g = grams; wk = weeks

Table 2 shows the mean and maximum sucking pressure observed during NNS in the EG and the CG. In the EG, the mean sucking pressure did not vary across the three measurements and reached a final value of 3.6 (± 0.9) mmHg. The mean sucking pressure in the CG was significantly lower (3.1 ± 1.1 mmHg) than that observed in the EG (p=0.02). A statistically significant difference between groups was also observed for the second measurement (3.6 ± 1.0 vs. 3.2 ± 0.9 mmHg for the EG and CG, respectively). The maximum NNS pressure was also higher in the EG as compared to the CG. Statistically significant group differences were observed for all maximum pressure values, including the overall mean.

**Table 2:** Suction pressure according to the presence or not of gustatory stimulus

	Presence of gustatory stimulus in NNS		
	EG N=30	CG N=30	p
MP1 (mmHg)	3.6 ± 1.0	3.2 ± 1.3	0.11
MP2 (mmHg)	3.6 ± 1.0	3.2 ± 0.9	0.005*
MP3 (mmHg)	3.6 ± 1.0	3.1 ± 1.6	0.08
Mean MP (mmHg)	3.6 ± 0.9	3.1 ± 1.1	0.02*
			
PMax1 (mmHg)	10.4 ± 2.2	9.1 ± 2.2	0.01*
MP2 (mmHg)	10.1 ± 2.2	8.3 ± 2.9	0.005*
PMax3 (mmHg)	9.7 ± 2.6	7.8 ± 3.1	0.007*
Mean PMax. (mmHg)	10.1 ± 2.2	8.4 ± 2.1	0.001*

* Significance by the Student's t-test; values expressed as mean and standard deviation

Caption: EG = experimental group; CG = control group; NNS = non-nutritive suction; MP = mean pressure; PMax. = maximum pressure

**Figure 4:**
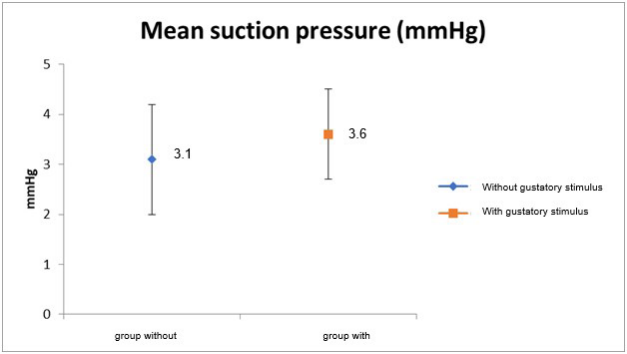
Mean suction pressure in groups of full-term newborn infants with (EG) and without gustatory stimulus (CG) during non-nutritive suction

**Figure 5:**
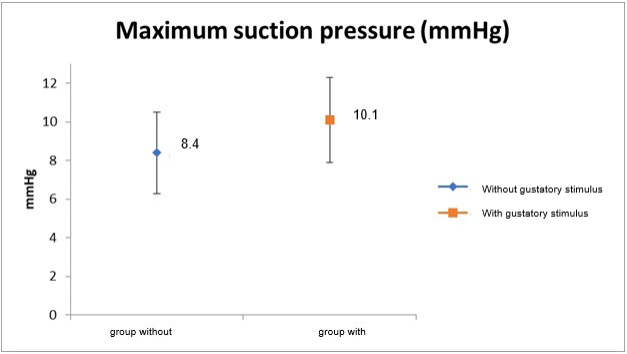
Maximum suction pressure in groups of full-term newborn infants with (EG) and without gustatory stimulus (CG) during non-nutritive suction

Figures 4 and 5 illustrate the mean and maximum NNS pressure in the EG and CG during NNS, respectively.

## DISCUSSION

The present study aimed to determine the influence of taste stimulation with colostrum on the quantitative assessment of sucking pressure during NNS in healthy infants. The present findings showed that wetting the pacifier with colostrum during the quantitative assessment of NNS led to higher pressure measurements, affecting both mean and maximum sucking pressure in the EG. This finding is in line with those of studies that reveal a faster suction response in newborns when a taste stimulus is introduced during NSS^(8-22)^.

The association of taste stimulation with NNS has been discussed in previous studies^(2,6,8,20,21)^. In some investigations where a sweet stimulus was used, the effects of this procedure have varied widely, ranging from increased amplitude and rhythm of suction bursts to a faster, safer, and more efficient transition to oral feeding^(8,14,20,22)^.

A possible explanation for the increase in sucking pressure after the introduction of the taste stimulus relates to the fact that the infant’s prior experience ingesting amniotic fluid in utero may have contributed to the development of their taste abilities^(21)^. Taste cells first appear in the 7th and 8th weeks of gestation, developing through weeks 13 to 15 and maturing by week 17^(25,26)^.

Swallowing appears at approximately the 12th week of gestation, but the coordination of sucking and swallowing is only achieved at gestation weeks 34-40. Amniotic fluid contains several nutrients with varying flavors, including fructose, glucose, fatty acids, lactic acid, and amino acids, as well as the flavors of foods consumed by the mother, all of which constitute the fetus’ first chemosensory experiences^(26)^.

It is important to note that the colostrum/mother’s milk is an important element of the sensory experience of breastfeeding and the early contact between mother and child, so that colostrum may be a strong inducer of the sucking response^(5,26)^. Colostrum is a yellowish, thick, and viscous liquid with a high quantity of immunobiological agents, lactoferrin, as well as anti- and pro-inflammatory cytokines, which can help modulate the infant’s inflammatory response^(27,28)^.

Studies of colostrum therapy have shown beneficial results, especially on the health of pre-term newborns. The oropharyngeal administration of colostrum for purposes other than nutritional support can stimulate immune development and improve the intestinal microbiota^(27,28)^.

Additionally, it is thought that colostrum therapy might have positive effects on the maturation of oral motor skills through the reduction of oral sensory deprivation, even in newborns who have not experienced breastfeeding. The stimulation of NNS, in turn, appears to support the stability and organization of the newborn, strengthening the stomatognathic system structures, especially the perioral muscles, allowing for adequate suction and swallowing pressures, required for oral feeding^(10,29,30)^.

In light of these findings, we conclude that the use of colostrum or mother’s milk during NNS, in both assessment and intervention procedures, may be a relevant tool for clinical practice in speech pathology. This practice may improve suction patterns and the oral-motor organization of the newborn for subsequent oral feeding.

Therefore, we believe that further longitudinal studies should be conducted to evaluate whether the effects of taste stimulation with colostrum, as described in this study, remain over time and/or have a positive influence on oral feeding performance.

It is important to note that though the quantitative method used to evaluate NNS constitutes a strength of this study, our quasi-experimental, non-randomized method may be viewed as a limitation, as it led to findings that are not as robust as those that could be obtained from a randomized experimental trial. The participating infants were also assessed at only a single time point in the day. We therefore suggest that additional longitudinal studies be conducted to quantify and observe the development of sucking patterns and oral-motor organization during oral feeding in newborns.

## CONCLUSION

The association of taste stimulation with NNS modified sucking pressure and appeared to enhance the oral abilities of newborns.
