# Supplementary material for: Influence of taste stimulation on sucking pressure in newborn infants at term
Source: Codas. 2022 Jan 12;34(3):e20210002. doi: 10.1590/2317-1782/20212021002 (PMC9769435; doi:10.1590/2317-1782/20212021002)
Supplement: Translation

# Translation: Influência do estímulo gustativo na pressão de sucção de recém-nascidos a termo

**Authors:** Fernanda Segala, Geovana de Paula Bolzan, Marlove Duarte Nascimento, Daniela da Silva Gonçalves, Amanda Melchior, Marcus Vinícius Marques de Moraes, Angela Regina Maciel Weinmann

**Affiliations:** 1 Programa de Pós-graduação em Distúrbios da Comunicação, Universidade Federal de Santa Maria – UFSM - Santa Maria (RS), Brasil.; 2 Departamento de Fonoaudiologia, Universidade Federal de Santa Maria – UFSM - Santa Maria (RS), Brasil.; 3 Departamento de Fisioterapia, Universidade Regional de Blumenau – FURB - Blumenau (SC), Brasil.; 4 Departamento de Fisioterapia, Hospital Universitário de Santa Maria, Universidade Federal de Santa Maria – UFSM - Santa Maria (RS), Brasil.; 5 Programa de Residência Multiprofissional, Hospital Universitário de Santa Maria, Universidade Federal de Santa Maria – UFSM - Santa Maria (RS), Brasil.; 6 Departamento de Fisioterapia, Universidade Regional de Blumenau – FURB - Blumenau (SC), Brasil.; 7 Departamento de Pediatria e Puericultura, Universidade Federal de Santa Maria – UFSM - Santa Maria (RS), Brasil.

**Keywords:** Comportamento de Sucção, Alimentação, Percepção Gustatória, Recém-Nascido, Leite Humano

## Abstract

**Objetivo:**

Verificar a influência de um estímulo gustativo na pressão de sucção, durante a sucção não nutritiva (SNN), em recém-nascidos a termo, saudáveis e com peso adequado à idade gestacional.

**Método:**

Estudo quase experimental do tipo ensaio clínico não randomizado com uma amostra de conveniência de 60 recém-nascidos (RN), 30 alocados no grupo estudo (GE) e 30 no grupo controle (GC). Os RN foram avaliados quanto à pressão de sucção, durante a SNN em chupeta. Para o GE foi adicionado estímulo gustativo à chupeta, umedecida com o colostro. O GC não recebeu nenhum estímulo, além da própria chupeta. As pressões média, mínima e máxima foram medidas com o equipamento S-Flex®.

**Resultados:**

O GE apresentou pressão média e máxima de sucção significativamente maiores do que o GC. Ainda, houve diferença estatisticamente significativa, entre os grupos, para a 2ª medida da pressão média de sucção.

**Conclusão:**

Os resultados demostraram que os RN do GE apresentaram pressões de sucção, média e máxima, significativamente maiores, quando comparados ao GC. A utilização de um estímulo gustativo associado à SNN modificou a pressão de sucção e parece potencializar as habilidades orais.

## INTRODUÇÃO

A sucção é um ato motor reflexo e fundamental para os recém-nascidos (RN), estando presente desde a vida intrauterina e tornando-se madura entre a 32ª a 34ª semana de idade gestacional^(1)^. Aproximadamente com 12 semanas gestacionais, o feto já demonstra movimentos de sucção, e com 20 semanas, abre e fecha a boca com ciclos de compressão organizados, e pausas regulares ^(2)^.

A função de sucção permite ao RN a ingestão de leite após seu nascimento. Porém, a sucção, pode também promover acalmia na realização de procedimentos dolorosos, favorecer o desenvolvimento do sistema estomatognático, bem como a percepção oral e a exploração do ambiente ^(3,4)^.

Além de uma boa sucção e coordenação harmônica entre sucção, deglutição e respiração ^(5)^ a organização comportamental do RN consiste em um dos indicadores de sucesso para a alimentação oral ^(6,7)^. A manutenção do estado de alerta durante a alimentação oral favorece ao RN o reconhecimento das várias sensações e estímulos orais, auditivos, vestibulares, táteis e cinestésicos, propiciando uma nutrição prazerosa, eficiente e segura ^(8-10)^.

A SNN faz parte da avaliação para prontidão alimentar dos RN e da intervenção motora oral, os quais são recursos primordiais para acelerar o processo de maturação das habilidades orais quando dificuldades são diagnosticadas, principalmente em prematuros ^(1,11-13)^. Nesse sentido, de avaliação das habilidades orais, pesquisadores propõem ferramentas que analisam tanto aspectos comportamentais, como sensoriais da SNN ^(3,9,14-17)^.

No contexto de avaliação quantitativa, o S-FLEX®, é um equipamento capaz de quantificar a pressão de SNN em RN ^(18)^_,_ sendo um recurso que oferece maior precisão na avaliação da sucção.

Esse equipamento apresenta características necessárias para o uso na rotina de avaliações, na área neonatal, por ser portátil, de simples manuseio e permitir uma higienização e esterilização em ambiente hospitalar. O S-FLEX® caracteriza-se pela segurança ambiental, já que não expõe o RN à corrente elétrica ou campo magnético.

A confiabilidade e reprodutibilidade do S-FLEX®, para a verificação da pressão de SNN em RN, já foi determinada, demonstrando sua aplicação e uso científico ^(19)^. Como precisamos contar com instrumentos mais objetivos e quantitativos, para avaliar a SNN, alguns estudos já estão sendo realizados com a utilização de estímulo gustativo para tal avaliação ^(6,8,20,21,22,)^.

O estímulo gustativo parece ser um fator influenciador no aumento da frequência e regularidade da sucção, aumentando a consistência no movimento da musculatura orbicular dos lábios, assim como do sistema sensório-motor ^(23)^. O rol de estímulos possíveis ao RN propiciaria uma maior sensação de prazer e satisfação, questões psicoafetivas e biológicas que promoveriam medidas mais efetivas e fidedignas do seu desempenho durante a alimentação ^(14,17,19,24)^.

No presente estudo abordamos a avaliação da SNN dos RN, com o uso do colostro como estímulo gustativo. Dessa forma, e com a expectativa de poder contribuir para a prática clínica dos profissionais envolvidos na avaliação da SNN dos RN, o objetivo desse estudo foi verificar a influência de um estímulo gustativo na pressão de sucção, durante a avaliação quantitativa da SNN, em RN a termo, saudáveis, antes da alta da maternidade.

## MÉTODO

### Desenho do estudo

Estudo quase experimental, tipo ensaio clínico não-randomizado.

### Amostra

A amostra foi de conveniência, sendo constituída por 60 RN internados na Unidade de Alojamento Conjunto do Hospital Universitário de Santa Maria (UFSM), no período de abril a novembro de 2019. Os RN constituíram dois grupos, formados de modo não aleatório: 30 neonatos no GE, que receberam estímulo gustativo durante a SNN e 30 neonatos no GC, que não receberam estímulo gustativo durante a SNN.

### Critérios de inclusão

Foram incluídos RN a termo, até o terceiro dia de vida, clinicamente estáveis, em aleitamento materno exclusivo e peso adequado para a idade gestacional.

### Critérios de exclusão

Foram excluídos RN com malformações congênitas de cabeça e pescoço, neurológicas e/ou cardíacas, síndromes genéticas, além de neonatos com instabilidade respiratória e/ou clínica no momento da avaliação.

### Aspectos éticos

O estudo foi aprovado no Comitê de Ética em Pesquisa (CEP) da Universidade Federal de Santa Maria, sob o número 11155312.7.00005346. Todos os participantes tiveram o Termo de Consentimento Livre e Esclarecido (TCLE) assinado pelos responsáveis.

### Procedimentos

Inicialmente foi realizada pesquisa aos prontuários de internação hospitalar para seleção dos RN, de acordo com os critérios de elegibilidade estabelecidos. Após a identificação dos RN, foi apresentado aos pais e/ou representantes legais o TCLE e, conforme o aceite dos mesmos, os RN foram incluídos na pesquisa. Em seguida, foram coletadas informações relativas ao nascimento como: dados de identificação, data e hora de nascimento, sexo, idade gestacional e peso ao nascer, Apgar de 1º e 5 º minuto, tipo de parto e adequação do crescimento intrauterino.

A avaliação da SNN, para medir a pressão de sucção (principal variável em estudo), foi realizada por um examinador treinado, com o RN em estado comportamental de alerta e tendo recebido a última mamada no seio materno no mínimo uma hora antes da avaliação.

Tanto no GE como no GC, a pressão de sucção foi mensurada utilizando o equipamento S-Flex® (Todmed). Considerou-se para análise o registro de um minuto efetivo de sucções. Para essa avaliação foi utilizada uma chupeta para captação da pressão de sucção. Todas as mães foram informadas quanto à necessidade de utilização da chupeta como recurso de avaliação e, quanto aos riscos de seu uso, além do momento do exame, principalmente no que tange ao desmame precoce e ao desenvolvimento do sistema estomatognático. Em vista disso, acredita-se que o uso desse recurso, durante a avaliação, não tenha favorecido a oferta de chupeta aos RN posteriormente pelas mães.

A coleta iniciou-se com a realização da ordenha manual de uma pequena quantidade de colostro que foi armazenado em um copo plástico. Essa etapa foi aplicada previamente, antes da avaliação, apenas para o GE. Da mesma forma, anteriormente ao início da avaliação propriamente dita, o RN precisou adaptar-se à chupeta (realizada com ambos os grupos (GE e GC). A única diferença entre os grupos foi que o GE teve o estímulo gustativo adicionado à chupeta (conforme descrito acima). Posteriormente, o RN era posicionado no colo do examinador, em posição de flexão com o tronco e cabeça elevados na linha média. Com a chupeta, o examinador estimulou gentilmente o reflexo de procura e a abertura da boca do neonato, introduzindo a chupeta na cavidade oral. A chupeta era sustentada com os dedos polegar e indicador do examinador, oferecendo estabilidade, sem gerar resistência. No caso de o RN empurrar a chupeta com a intenção de soltá-la, esta era liberada pelo examinador e reintroduzida na cavidade oral, para a retomada da avaliação. Foi permitida a sucção da chupeta até o final da mensuração. Não foi estabelecido um número máximo de vezes que a chupeta poderia ser reintroduzida na cavidade oral do neonato e, esse dado, também, não foi considerado no momento da análise dos dados. Porém, ao menor sinal de estresse do RN, como choro ou fadiga, a coleta de dados era interrompida e, retomada apenas quando ele demonstrasse sinais de conforto. O início da gravação da medida de pressão da SNN ocorria somente após a adaptação do RN com a forma e textura da chupeta. Quanto a adição do estímulo gustativo à chupeta, o examinador mergulhou o dedo enluvado no colostro e passou seu dedo molhado ao redor da chupeta, sem fazer medição do volume de colostro, pois a quantidade necessária para umedecer a chupeta deveria ser muito pequena, cerca de uma gota de colostro. A chupeta umedecida foi imediatamente oferecida ao RN. Assim que o RN se adaptou com a chupeta e iniciou os movimentos de sucção efetivos, era solicitado ao avaliador auxiliar o acionamento do *display* para o registro da pressão de sucção. A chupeta era considerada solta quando o display do equipamento marcava zero. Considerando que o equipamento S-FLEX® possui um tempo de memória máximo de 22 segundos, foram estabelecidas três determinações ou medidas da pressão de sucção, para cada RN, a fim de totalizarem um minuto de registro. O tempo máximo dispensado para a avaliação foi de 10 minutos. Por fim, para o registro e gravação das medidas, o examinador auxiliar realizava a análise de três medidas registradas e àquelas com melhor qualidade do traçado, foram escolhidas para serem salvas para posterior análise.

Quanto a higienização dos materiais, ao término das avaliações, todo material utilizado era higienizado e encaminhado para autoclavagem, seguindo as normas e rotina de esterilização do hospital.

A Figura 1 ilustra o procedimento de coleta, com o posicionamento do RN no colo do examinador e a sustentação da chupeta na cavidade oral do neonato.

**Figura 1:** Procedimento de coleta dos dados com um RN, seu posicionamento e sustentação da chupeta

O instrumento utilizado para medir a pressão de sucção foi o S-Flex® da empresa nacional Todmed. Esse equipamento registra a pressão de SNN de RN, tanto na forma gráfica como numérica. O equipamento possui tecnologia *touch screen* e possui interface de comunicação com o computador pessoal através de *software* em ambiente *Windows®*, porta dispositivo *USB* (*pendrives* ou disco externo) com uma fonte externa de voltagem de 127 a 240 Volts para carregamento da bateria, a qual tem funcionamento de 4 horas. As mensurações são feitas em unidades físicas com registros em mmHg e g/cm^2^.

Esse equipamento é composto de um bico, com orifício na extremidade, acoplado a um aro confeccionado no formato côncavo e anatômico, com um sensor de pressão. A conexão da chupeta ao aparelho é feita por um tubo com espessura de 1,25mm e comprimento de 1,5m. O bico que faz parte desse sistema, confeccionado em silicone e em formato ortodôntico, é extraído de uma chupeta modelo Oral Fit, tamanho 1 da marca comercial NUK®, indicado para RN de 0 a 6 meses de idade. Na extremidade desse bico foi aberto um orifício com ponta diamantada 1014 (*KGS*). Os materiais e componentes são de uso individual e permitem a higienização e esterilização por autoclavagem adequadas, sem risco de contaminação cruzada. O tubo de conexão com a chupeta e os outros componentes satisfazem os requisitos de biocompatibilidade e segurança elétrica, preenchendo todos os critérios de biossegurança hospitalar. Ainda se caracteriza por ser leve, portátil e permitir a captura dos dados em diferentes lugares. Abaixo apresentamos a Figura 2 e Figura 3 com a representação do equipamento S-FLEX® e os seus componentes, respectivamente.

**Figura 2:** Equipamento S-FLEX®

Legenda: (1) bico ortodôntico; (2) arco côncavo e anatômico; (3) sensor de pressão; (4) tubo 1,25 mm × 1,5m

**Figura 3:** Componentes do equipamento S-FLEX®

### Análise dos dados

Os dados obtidos foram transferidos e tabulados no programa *Excel*, sendo analisados através do *software* STATA 10. As variáveis contínuas foram expressas em médias e desvio padrão e as variáveis categóricas em percentuais. A normalidade das variáveis analisadas foi testada pelo teste de Shapiro-Wilk. Os grupos foram comparados entre si através do teste t-Student (variáveis contínuas) e teste do Qui-quadrado de Pearson (variáveis categóricas). Foi considerado significativo um nível de p0,05, em todas as análises.

## RESULTADOS

As características do GE e GC estão apresentadas na Tabela 1. No GE a média da idade gestacional e do peso ao nascer foi de 39 (±1,1) semanas e 3141 (± 262) gramas, e no GC, de 38,7 (±1,10) semanas e 3132 (±414) gramas, respectivamente. Os RN dos dois grupos foram classificados como adequados em peso para sua idade gestacional ao nascer. Quanto ao sexo, no GE, 53,3% eram meninas e 46,7% meninos. No GC, observou-se um maior percentual de meninos (60 X 40%, para meninos e meninas, respectivamente). Quanto a vitalidade ao nascer, em ambos os grupos, a média do Apgar, tanto no primeiro minuto, quanto no quinto minuto de vida foi superior a sete. Embora a constituição dos grupos tenha ocorrido de forma não aleatória, não houve diferença estatisticamente significativa em todas as características avaliadas.

**Tabela 1:** Características dos recém-nascidos a termo estudados

	Presença de estímulo gustativo na SNN		
	GE N=30	GC N=30	p
Peso ao nascer (g)	3141 ± 262	3132 ± 414	0,46^1^
IG ao nascer (sem)	39 ± 1,1	38,7 ± 1,1	0,17^1^
Sexo (% (N))			
Masculino	46,7 (14)	60 (18)	0,30^2^
Feminino	53,3 (16)	40 (12)	
Tipo parto			
Vaginal	53,3 (16)	73,3 (22)	0,10^2^
Cesáreo	46,7 (14)	26,7 (8)	
Apgar 1º minuto	8,6 ± 1,3	8,8 ± 1,3	0,22^1^
Apgar 5º minuto	9,7 ± 0,5	9,8 ± 0,4	0,19^1^
Dias de vida avaliação	2 ± 1,26	1,67 ± 0,71	0,261^1^

1 Teste t-Student;

2 Qui-quadrado de Pearson

Legenda: GE = grupo estudo; GC = grupo controle; SNN = sucção não-nutritiva; IG = idade gestacional; g = gramas; sem = semanas

A Tabela 2 apresenta os valores da pressão média e máxima de sucção, durante a sucção não nutritiva dos participantes, tanto no GE como no GC. No GE, a pressão média de sucção não variou nas três aferições, sendo a pressão média de sucção final de 3,6 (±0,9) mmHg. Já GC, a pressão média de sucção final foi significativamente menor (3,1 ±1,1 mmHg), quando comparada à do GE (p=0,02). Diferença estatisticamente significativa, entre os grupos, também foi observada para a 2ª medida (3,6 ±1,0 X 3,2 ±0,9 mmHg para os GE e GC, respectivamente). Quanto à pressão máxima de sucção, esta foi maior no GE, quando comparada ao GC. Houve diferença estatisticamente significativa entre os grupos para todas as medidas de pressão máxima, incluindo a média final.

**Tabela 2:** Pressão de sucção conforme a presença ou não do estímulo gustativo

	Presença de estímulo gustativo na SNN		
	GE N=30	GC N=30	p
PM1 (mmHg)	3,6 ± 1,0	3,2 ± 1,3	0,11
PM2 (mmHg)	3,6 ± 1,0	3,2 ± 0,9	0,005*
PM3 (mmHg)	3,6 ± 1,0	3,1 ± 1,6	0,08
Média PM (mmHg)	3,6 ± 0,9	3,1 ± 1,1	0,02*
			
PMáx1 (mmHg)	10,4 ± 2,2	9,1 ± 2,2	0,01*
PMáx2 (mmHg)	10,1 ± 2,2	8,3 ± 2,9	0,005*
PMáx3 (mmHg)	9,7 ± 2,6	7,8 ± 3,1	0,007*
Média PMáx. (mmHg)	10,1 ± 2,2	8,4 ± 2,1	0,001*

* Significância pelo Teste t-Student; valores expressos em média e desvio padrão

Legenda: GE = grupo estudo; GC = grupo controle; SNN = sucção não-nutritiva; PM = pressão média; PMáx = pressão máxima

As Figuras 4 e 5 ilustram as pressões média e máxima de sucção nos GE e GC, durante a SNN, respectivamente.

**Figura 4:** Pressão Média de sucção nos grupos de RN a termo com (GE) e sem estímulo gustativo (GC) durante a SNN

**Figura 5:** Pressão Máxima de sucção nos grupos de RN a termo com (GE) e sem estímulo gustativo (GC) durante a SNN

## DISCUSSÃO

O presente estudo objetivou determinar a influência do uso do colostro, como estímulo gustativo, na pressão de sucção, durante a avaliação quantitativa da SNN, em RN saudáveis. Os resultados encontrados na pesquisa mostraram que, umedecer a chupeta com colostro, durante a avaliação quantitativa da SNN, produziu maiores valores de pressões de sucção, tanto média como máxima, nos RN do GE. Esse achado vai ao encontro de pesquisas que evidenciaram respostas de sucção mais rápidas dos RN quando adicionado um estímulo gustativo durante a SNN ^(8-22)^.

A utilização de estímulos gustativos associados à SNN vem sendo discutida na literatura ^(2,6,8,20,21)^. Porém, em alguns estudos, o estímulo gustativo é realizado com sabor adocicado e os autores verificaram que quando esse sabor é percebido, as respostas dos RN vão desde o aumento na amplitude e ritmo de sucção, até uma transição para alimentação oral mais rápida, segura e eficiente ^(8,14,20,22)^.

Uma possível explicação para uma melhor resposta de sucção, mediante o estímulo gustativo, poderia ser a partir do pressuposto de que o RN já tem experiência prévia da deglutição de líquido amniótico, na sua vida intrauterina, tendo a capacidade gustativa desenvolvida ^(21)^. As células gustativas começam a se formar a partir da 7ª e 8ª semanas de gestação, até por volta da 13ª a 15ª semanas, já estando maduras na 17ª semana ^(25,26)^.

A deglutição fetal inicia aproximadamente na 12ª semana de gestação, mas a sucção e deglutição atingem a coordenação harmônica e satisfatória em torno da 34ª a 40ª semanas de gestação. Na composição do líquido amniótico encontram-se vários nutrientes com sabores particulares, como: frutose, glicose, ácidos graxos, ácido lático e aminoácidos, bem como os sabores dos alimentos consumidos pela mãe, e que serão as primeiras experiências quimiossensoriais do feto ^(26)^.

Vale dizer, que o colostro/leite materno poderia aproximar o neonato da experiência sensorial da amamentação, do contato precoce da mãe com o RN e sugere, então, que o colostro pode ser um forte eliciador de sucção ^(5,26)^. O colostro caracteriza-se por ser um líquido amarelado, espesso e viscoso, com uma quantidade de imunobiológicos, lactoferrina, citocinas anti e pró-inflamatórias, que em contato com a mucosa oral são capazes de modular a resposta inflamatória dos RN ^(27,28)^.

Estudos sobre a colostroterapia já mostram resultados benéficos, principalmente para manter a saúde dos RN prematuros. A administração orofaríngea do colostro, sem função nutricional, é uma técnica que estimula o desenvolvimento da imunidade e melhora a microbiota intestinal ^(27,28)^.

Contudo, acredita-se na perspectiva de outros benefícios adicionais da colostroterapia, relacionados à maturação das habilidades motoras orais, por meio da redução da privação sensorial oral, mesmo em RN que ainda não tiveram a experiência de aleitamento materno. A estimulação da SNN, por sua vez, parece fornecer a estabilidade e a organização do RN, fortalecendo as estruturas do sistema estomatognático, especialmente a musculatura peri-oral, proporcionando uma adequada pressão de sucção e deglutição, necessárias à alimentação oral ^(10,29,30)^.

Com base nos achados desse estudo, considera-se que a utilização do colostro ou do leite materno durante a SNN, seja para procedimentos de avaliação ou intervenção, poderá ser útil na prática clínica fonoaudiológica. Principalmente, por favorecer a melhora no padrão de sucção do neonato e, dessa forma, propiciar uma melhor organização motora oral do RN para a alimentação oral.

Assim, vislumbra-se que novos estudos longitudinais possam ser realizados, a fim de avaliarem se os efeitos do estímulo gustativo com colostro, referidos nesse estudo, mantém-se ao longo do tempo e/ou se são capazes de promover efeitos positivos sobre a performance alimentar oral.

Cabe salientar, que apesar da vantagem de se utilizar nesse estudo um método quantitativo para avaliação da SNN, sua metodologia quase-experimental, não-randomizado, representa uma limitação, já que a evidência dos resultados obtidos não tem o mesmo poder de um estudo experimental randomizado. O tempo de observação dos grupos de bebês foi limitado a apenas um momento ao longo do dia. Assim, sugere-se, que sejam realizados mais estudos longitudinais, capazes de quantificar e observar o melhor desenvolvimento do padrão de sucção e organização motora-oral do neonato para a alimentação oral.

## CONCLUSÃO

A utilização de um estímulo gustativo associado à SNN modificou a pressão de sucção, e parece potencializar as habilidades orais de RN.
